# Platelet-derived microRNA-223 attenuates TNF-α induced monocytes adhesion to arterial endothelium by targeting ICAM-1 in Kawasaki disease

**DOI:** 10.3389/fimmu.2022.922868

**Published:** 2022-08-02

**Authors:** Manli Guo, Shunyang Fan, Qian Chen, Cuiping Jia, Miaoyun Qiu, Yun Bu, Wai Ho Tang, Yuan Zhang

**Affiliations:** ^1^ Institute of Pediatrics, Guangzhou Women and Children’s Medical Centre, Guangzhou Medical University, Guangzhou, China; ^2^ Heart Center, The Third Affiliated Hospital of Zhengzhou University, Zhengzhou, China

**Keywords:** Kawasaki disease, endothelial cells, miR-223, platelets, ICAM-1

## Abstract

**Background:**

Kawasaki disease (KD) is an acute vasculitis that may result in permanent coronary artery damage with unknown etiology. Endothelial cell (EC) dysfunction and platelet hyperactivity are the hallmarks of KD. Platelets are involved in the development of endothelial dysfunction. MiR-223 transferred by platelet microparticles (PMPs) has been found to involve in the functional regulation of endothelial cells in sepsis. However, the role of platelet-derived miR-223 in endothelial dysfunction has not yet been investigated in KD.

**Objectives:**

We seek to investigate the role of platelet-derived miR-223 in endothelial dysfunction of KD vasculopathy.

**Methods and results:**

Forty-five acute KD patients and 45 matched controls were randomly recruited in the study. When co-cultured with human coronary artery endothelial cells (HCAECs), KD platelets with higher levels of miR-223 were incorporated into HCAECs, resulting in the horizontal transfer of miR-223. Using KD platelets, PMPs, and platelet-releasate from the same amount of blood co-cultured with HCAECs, we found the increased expression of miR-223 in HCAECs was primarily derived from KD platelets, rather than PMPs or free miRNAs from platelet- releasate. KD platelet-derived miR-223 attenuated TNF-α induced intercellular cell adhesion molecule-1 (ICAM-1) expression in HCAECs. KD platelet-derived miR-223 also suppressed the monocyte adhesion to HCAECs. *In vivo*, platelet-specific miR-223 knockout (PF4-cre: miR-223^flox/flox^) C57BL/6 mice and miR-223^flox/flox^ C57BL/6 mice were used. Using *Lactobacillus* casei cell wall extract (LCWE) to establish KD murine model, we showed that in LCWE-injected PF4-cre: miR-223^flox/flox^ mice, deficiency of platelet-miR-223 exacerbates the medial thickening of the abdominal aorta, increased ICAM-1 expression with concomitant CD45^+^ inflammatory cells infiltration into the endothelium compared to LCWE-injected miR-223^flox/flox^ mice.

**Conclusions:**

The horizontal transfer of platelet-derived miR-223 suppresses the expression of ICAM-1 in HCAECs, which at least in part attenuates leukocyte adhesion, thereby reducing endothelial damage in KD vasculitis

## Introduction

Kawasaki disease (KD) is a children’s acute systemic vasculitis of unknown etiology, which mainly impacts small and medium arteries, and can cause coronary aneurysms (1). In KD, a series of pathological changes occur, including endothelial injury, platelet hyperreactivity, infiltration of inflammatory cells, and vascular smooth muscle cell (VSMC) differentiation, although the relationship between these cells remains unclear (2). The current treatment for KD is the timely use of high-dose intravenous immunoglobulin (IVIG) and aspirin, for anti-inflammation and anti-platelet aggregation (3). Nevertheless, the occurrence of coronary aneurysms cannot be completely avoided, 4% of treated patients will still develop a coronary aneurysm (4). Therefore, understanding the pathogenesis of coronary arterial aneurysms will provide more effective therapy for KD patients.

The autopsy revealed that the onset of KD vasculitis was first in the luminal endothelium (1), which was characterized by numerous infiltration of inflammatory cells, edema and necrosis of endothelial cells (5). Previous studies have shown that infiltration of inflammatory cells upregulates the endothelial cell adhesion molecules (such as ICAM-1) by secreting cytokines (6), leading to the adhesion and chemotaxis of leukocytes (e.g. monocytes), further promoting endothelial damage (7, 8). Endothelial damage promotes platelet activation (9) and releases cytokines (e.g. platelet-derived growth factor-BB, PDGF-BB), which promote VSMC dedifferentiation (10). VSMC dedifferentiation plays a critical role in the coronary pathology of KD by promoting medial damage, thickening, and aneurysm formation (1). In addition, activated platelets can induce inflammatory responses in endothelial cells by releasing pro-inflammatory factors (e.g. interleukin-1β) or sheared CD40 ligands, leading to endothelial damage (11–15). Platelets are actively involved in the development of KD-induced vascular injury.

Platelets are non-nucleated cells formed by dissociation of the cytoplasm of mature megakaryocytes in the bone marrow. Early studies showed that platelets mainly play a role in thrombosis and homeostasis. Recently, accumulating evidence has shown that platelets contain a large number of miRNAs (16), as well as the subcellular mechanism of *de novo* protein synthesis (17). MicroRNA (miRNA) is a short non-coding RNA that inhibits the expression of target genes by interacting with the 3 ‘UTR of target mRNA (18). A previous study showed that miRNAs, such as miR-223, miR-337, and miR-199a, were differentially expressed in platelets in KD (19). MiR-223, which is abundantly expressed in platelets (20) has been found to participate in the pathological process of cardiovascular diseases (19, 21–23). For example, in atherosclerosis, miR-223 contributes to cholesterol transport, biosynthesis, and efflux by targeting several genes associated with cholesterol transport, biosynthesis, and efflux, and subsequently relieves hyperlipidemia (24). Recent studies have shown that miR-223 transferred by PMPs or platelets is involved in the functional regulation of recipient cells. In sepsis, PMPs-derived miR-223 has been shown to reduce ICAM-1-Dependent Vascular Inflammation (25). PMPs-derived miR-223 inhibited insulin-like growth factor receptor (IGF-1R), which promoted advanced glycation end products (AGEs)-induced endothelial cell apoptosis (26). In addition, platelet-derived miR-223 regulates VSMC dedifferentiation (27). Deficiency of platelet miR-223 in KD patients with aneurysms contributes to coronary artery pathology as platelet uptake fails to suppress VSMC dedifferentiation (19). Therefore, we hypothesize that platelet-derived miR-223 regulates endothelial dysfunction in KD vasculopathy.

In the present study, we now demonstrate that KD platelets with higher level of miR-223 were incorporated into HCAECs, resulting in the horizontal transfer of miR-223. KD platelet-derived miR-223 attenuates tumor necrosis factor-alpha (TNF-α) induced expression of ICAM-1 in HCAECs. In LCWE-injected PF4-cre: miR-223^flox/flox^ mice, deficiency of platelet-miR-223 exacerbates the medial thickening of the abdominal aorta, increased ICAM-1 expression, with concomitant CD45^+^ inflammatory cells infiltration into the endothelium compared to LCWE-injected miR-223^flox/flox^ mice. Thus, platelet-derived miR-223 contributes to attenuating the endothelial pathology of KD.

## Methods

### Human subjects

A total of 45 children with acute Kawasaki disease (31 males and 14 females, mean age, 48months) and 45 healthy children (23 males and 22 females, mean age, 46 months) were randomly recruited to the study as previously described (19) (Guangzhou Women and Children Medical Center Human Investigation Committee, No. 2017102710). The diagnosis of KD meets the criteria developed in 2004 by the American Heart Association. Written informed consent was obtained from the patient’s guardian or the adolescents themselves.

### Animals

The animal experiments were conducted under the authorization of the Animal Care and Use Committee of Guangzhou Medical University, China (2019-384). The PF4-cre: miR-223^flox/flox^ mice were created using PF4-cre mice [C57BL/6-Tg (PF4-icre) Q3Rsko/J, stock 008535] and miR-223^flox/flox^ mice(C57BL/6-miR-223^em1(flox)Smoc^). PF4-cre mice were purchased from Jackson Laboratory. MiR-223^flox/flox^ mice were obtained from Shanghai Model Organisms Center, Inc, China. The PF4-cre: miR-223^flox/flox^ mice were described below as PF4-miR-223 KO mice, miR-223^flox/flox^ mice were described below as Floxed control mice.

### Platelet and PMP purification

The whole blood was drawn from human subjects and centrifuged at 250g for 20 mins at room temperature to obtain platelet-rich plasma (PRP). The PRP was shifted to a new collection tube, followed by second centrifugation to absolutely spin down the residual blood cells. After treating with 100nM Prostaglandin E1 (PGE1, Sigma), the supernatant was centrifuged at 1000g for 5 mins to segregate plasma and platelets. The platelets were resuspended to 10^8^ platelets/ml in HEPES-Tyrode’s buffer. Platelet markers (CD41>95% positive) were used to determine the purity of platelets. The extraction method of platelet microparticles (PMPs) is described in previous studies (28). PMPs were extracted from the same amount of blood. Briefly, the platelet resuspension was slowly shaken at 10 rpm at room temperature for 4 hours. Next, platelets were centrifuged at 1000g for 5 mins. The supernatant was then centrifuged for 90 mins at 20,000 g at 18°C to obtain PMPs precipitates, and be resuspended in HEPES Tyrode’s buffer in the same volume as the platelets described above. Platelet markers (CD41>90% positive) were used to determine the purity of PMPs. The supernatant containing platelet-releasate was collected and stored at -80°C.

### Cell culture

Human coronary artery endothelial cells (HCAECs) were purchased from Lonza, USA, and cultured in the EBM-2 basal medium supplemented with an EGM-2 SingleQuots kit (CC-3162, Lonza, USA) containing 2% FBS and fibroblast growth factor (FGF), vascular endothelium growth factor (VEGF), human endothelial growth factor (EGF), hydrocortisone, insulin-like growth factor-1(IGF-1), ascorbic acid, heparin. For all experiments, HCAECs were used between passages 5 to 7. The promonocytic leukemia cell line (THP-1) was purchased from FuHeng biology, China, and cultured in the RPMI1640 medium supplemented with 10% fetal bovine serum (FBS). Cells were cultured at 37°C in a humidified 5% CO2 atmosphere with the medium replaced every 48 hours. In subsequent experiments, platelets, PMPs, and the supernatant were added 100μl per dish and co-cultured with HCAECs for 24 hours. Transwell chambers with a pore size of 1μm were used, which only allowed PMPs to pass through. For cell transfection experiments, the agomiR-223/agomiR-NC or siRNA-Dicer1 (si-Dicer, GenePharma, China) were transiently transfected at a concentration of 100nM using Lipofectamine RNAiMAX (Invitrogen reagent, USA) for 24 or 48 hours. Cells were harvested for subsequent experiments. The sequences of agomiRNA or siRNA were listed in **Supplementary Table 1**.

### Enzyme-linked immunosorbent assay (ELISA)

The levels of platelet factor 4 (PF4), β-thromboglobulin (β-TG), tumor necrosis factor-alpha (TNF-α) in human plasma were determined by platelet factor 4, PF4 ELISA Kit (CUSABIO, CSB-E07882h), β-thromboglobulin, β-TG ELISA Kit (CUSABIO, CSB-E07886h), Tumor necrosis factor-alpha, TNF-α ELISA Kit (CUSABIO, CSB-E04740h). One hundred microliters of plasma were incubated with the captured antibodies and secondary antibodies according to the instructions. The intensity was measured at 450nm and the reference was measured at 540 nm. The optical imperfections in the plate were corrected by subtracting the reading of 540 nm from 450nm. A standard curve was drawn according to the instructions to calculate the protein content of interest in each sample.

### Flow cytometry

Cell expression of P-selectin (CD62P) was analyzed according to the instructions. Briefly, platelets (10^8^ platelets/ml) suspended with 100μl HBSS buffer were incubated with PE anti-human CD62P antibody (BD, USA) at room temperature for 15 mins. After washing, the platelets and ECs were resuspended in HEPES-Tyrode’s buffer and analyzed by BD FACSCanto™.

### RNA isolation and quantitative RT-PCR

MiRNAs from platelets or HCAECs were purified using a miRNeasy mini kit (QIAGEN). According to the instructions, the extracted RNAs were reverse-transcribed using a PrimeScript™ RT kit (Takara) and analyzed by RT-qPCR using a Hairpin-it™ miRNA qPCR quantitative kit (GenePharma). Real-time quantitative PCR (SYBR-green, TAKARA) assays were performed with an Applied Biosystems Q6 Fast Real-Time PCR System sequencer detector. Expression was normalized to the expression of small nuclear RNA U6 (snU6) or the human GAPDH housekeeping gene. The primer sequences used in RT-qPCR were listed in **Supplementary Table 2**.

### Immunofluorescence

HCAECs were seeded into a glass-bottom culture dish at a density of 1.0×10^5^ per dish. Human platelets were incubated with Cell Tracker™ Green CMFDA (Life Technology) at a concentration of 1μM in darkness for 30 mins at 37°C. After washing at least three times, the platelets were resuspended in HEPES-Tyrode’s buffer at a concentration of 1.0×10^8^/ml. HCAECs were co-cultured with the platelets at a 1:100 ratio. After washing with PBS buffer, cells were fixed and incubated with primary antibodies: anti-CD31 (1:100, Abcam, ab9498) or anti-ICAM-1(1:200, Abcam, ab282575) overnight at 4°C. The next day, after incubating with the secondary antibody: Alexa Fluor 594-conjugated IgG antibody (1:200, Abcam, ab150116), Fluor 488-conjugated IgG antibody (1:200, Abcam, ab150077) at 37°C for 2 hours, the cells were stained with Hoechst to visualize the nucleus. Immunofluorescence images were captured by a confocal microscope (Leica SP8). In each independent experiment, three representative images were taken for calculating the mean fluorescence intensity by ImageJ software, and used as one independent data.

For tissue sections, the slides were incubated in PBS containing 5% normal goat serum, 5% bovine serum albumin, and the primary antibody mixture at 4°C overnight. The primary antibodies used were as follows: anti-ICAM-1 (1:200, Abcam, ab222736), anti-CD31 (1:100, Abcam, ab56299), anti-CD45(1:100, Proteintech, 60287). After incubating with each primary antibody, the slides were washed in PBS and incubated with Alexa Fluor 488-conjugated IgG secondary antibody (1:200, Abcam, ab150077) or Alexa Fluor 594-conjugated IgG secondary antibody (1:200, Abcam, ab150160) at 37°C for 2 hours. DAPI staining was performed to visualize the cell nucleus. Immunofluorescence images were taken with Leica SP8 confocal microscopy (Leica).

### Transmission electron microscopy (TEM)

HCAECs were plated in 65mm Petri dishes at a density of 5×10^5^ cells per dish. KD platelets were added at a ratio of 1:100 in co-culture with HCAECs for 24 hours. After washing with PBS twice, 1ml electron microscope fixative solution was added. Cells were scraped down with cell curettage and fixed at 4°C for more than 2 hours. After washing three times with 0.1M phosphoric acid buffer, cells were fixed again with 1% osmium tetroxide for 2 hours. After repeated washing with 0.1M sodium cacodylate buffer, cells were dehydrated in 30%-50%-70%-80%-95%-100%-100% ethanol successively. After uranyl acetate and lead citrate staining, ultrathin sections were examined with the transmission electron microscope FEI CM100 (Japan Electron Optics Laboratory)

### Monocyte adhesion assay

HCAECs (1.0×10^5^ cells per dish) were seeded and transfected as described above. As previously described (29), HCAECs were co-cultured with HC/KD platelets or transfected with agomiR-NC/agomiR-223 for 48 hours. After washing twice with PBS, cells were treated with TNF-α (2 ng/ml) for 4 hours and co-cultured with THP-1 (1.0×10^5^) cells labeled with Cell Tracker™ Green CMFDA (Life Technology, 1μM, 30mins, 37°C) for 2 hours. HCAECs were gently washed with PBS and fixed with the 4% paraformaldehyde solution for 10mins, then incubated with anti-CD31 (1:100, Abcam, ab9498) at 4°C overnight. After incubating with the Alexa Fluor 594-conjugated IgG antibody (1:200, Abcam, ab150116) at 37°C for 2 hours, cells were stained with Hoechst to visualize the nucleus. Three images per sample were captured by confocal microscope (Leica SP8), and the number of adhering monocytes was calculated by ImageJ software. The average number of adhered monocytes per sample was used as one individual data for statistical analysis.

### Western blotting

Total protein was extracted using Radio Immunoprecipitation Assay (RIPA) lysis buffer (Beyotime, China). Protein lysates were separated on 10% SDS-PAGE gels and transferred to PVDF membranes. After blocking with 5% skimmed milk, the membranes were incubated with primary antibodies: anti-ICAM-1(1:1000, Abcam, ab282575), anti-α-tubulin (1:2000, Abcam, ab52866) overnight at 4°C, and subsequently incubated with the secondary antibodies for an hour. The protein bands were detected using an ECL chemiluminescence kit (Beyotime, China) and quantified by ImageLab software (USA).

### Ago2 immunoprecipitation

HCAECs were seeded in 65mm culture dishes (5.0×10^5^ cells per dish) and transfected with agomiR-223/agomiR-NC at a concentration of 100nM for 48 hours. After harvested with RIP lysis buffer supplemented with protease and RNase inhibitors, the cell lysates were immunoprecipitated with Ago2 (SAB4200085, SIGMA) or isotype IgG antibody-coated beads (Merck Millipore). The immunoprecipitated RNA was extracted and reverse transcribed as described in the RNA isolation and quantitative RT-PCR section.

### LCWE preparation

The Lactobacillus casei cell wall extract (LCWE) was prepared as previously described (19, 30). Briefly, Lactobacillus casei (ATCC 11578) was grown in Lactobacillus MRS Broth (Difco) for 48 hours, harvested and washed with PBS. After disturbing by 2 volumes of 4% SDS/PBS at 37°C overnight, the cell wall fragment was washed 8 times with PBS to remove any SDS residue, and incubated with RNase, DNase, and trypsin. The cell wall fragment was sonicated for 2 hours at a pulse setting of 5.0 (10-s pulse/5-s pause) with cooling in an ice bath. After sonication, the cell wall fragments were spun for 20 mins at 12,000 rpm, 4°C. The supernatant was observed by centrifugation at 38,000 rpm, 4°C for 1 hour. The concentration of LCWE was determined based on the rhamnose content by using a colorimetric phenol-sulfuric assay.

### KD murine model

KD murine model was prepared as previously described (19). Four hundred micrograms of LCWE or PBS were injected intraperitoneally into 4-week-old mice. The mice were sacrificed by isoflurane gaseous anesthesia two weeks after LCWE injection. The whole blood was taken directly from the right ventricle before perfusion. The abdominal aorta was taken out and embedded in the optimal cutting temperature (OCT) complex for histological analysis. The blood sample was then collected for plasma and platelet precipitation as described in Human samples.

### Genotyping

Tails were clipped from mice 1-2 weeks after birth, about 2mm. Total DNA was extracted from individual tissues using the DNeasy Blood and Tissue Extraction Kit (QIAGEN). DNA pellets dissolved in water were used for PCR analysis. Primers were designed to detect alleles of WT, Floxed ctrl and recombinant PF4-miR-223 KO mice under standard PCR conditions. Primer sequences were listed in **Supplementary Table 3**.

### HE staining

The frozen sections were dried at 37°C for 30 mins and placed in PBS for 10 mins. After being immersed in hematoxylin for 4 mins, hydrochloric acid and alcohol for 1 sec, and eosin for 1 min, the sections were fixed with neutral gum and detected by fluorescence microscope (Leica DM4). The areas of thickened medial layer were compared between groups and subjected to statistical analysis for significance.

### Statistical analysis

Statistical analysis was performed using GraphPad Prism v.8.0.2 (USA). The Shapiro-Wilk normality test was used to determine the data distribution. For normally distributed data, values were presented as Mean ± SD. For two sets of data, an unpaired two-tailed Student’s *t-t*est was used for comparison. For multiple sets of data, analysis of One-way ANOVA was used, followed by Tukey’s multiple comparisons. For non-normally distributed data, these values were expressed as Median ± interquartile range (IQR). For two sets of data analysis, the Mann-Whitney test was used, while Kruskal-Wallis and Dunn’s multiple were used for multiple sets of data analysis. A difference of *P*<0.05 was considered statistically significant.

## Results

### KD platelets are hyperactive with increased expression of miR-223

**Figure 1:**
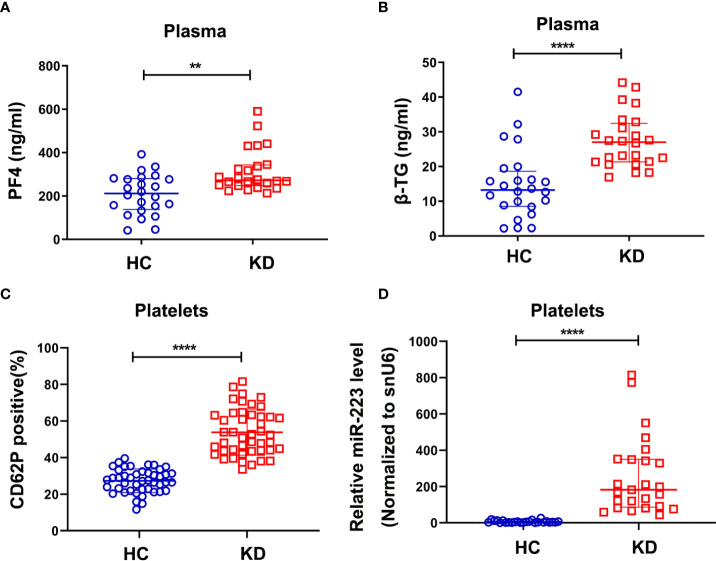
KD platelets were hyperactive and exhibit higher expression of miR-223. Plasma levels of PF4 **(A)** and β-TG **(B)** in HC and KD groups (HC: n = 24, KD: n = 24). Data are presented as median ± IQR, Mann Whitney test. **(C)** Surface expression of P-selectin (CD62P) in platelets isolated from HC and KD groups (HC: n = 45, KD: n = 45). Data are presented as mean ± SD, Unpaired *t-*test. **(D)** Relative expression of miR-223 in platelets isolated from HC and KD groups (HC: n=25, KD: n=25). Data are presented as median ± IQR, Mann Whitney test. ^**^ *P *< 0.01, ^****^ *P *< 0.0001. ns means no statistical difference.

As is known that KD is an acute febrile childhood disease characterized by multisystem vasculitis and platelets play an important role in the regulation of inflammatory diseases. We found that plasma levels of platelet factor 4 (PF4) and β-thromboglobulin (β-TG), as markers for platelet activation, were significantly increased in the KD group compared with the HC group **(** **Figures 1A, B** **)**. Meanwhile, the surface expression of p-selectin (CD62P) in KD platelets was 2~3 fold higher than those in HC platelets **(** **Figure 1C** **)**. These results indicated that KD platelets were highly activated. Our previous study using Genome-wide miRNA sequencing (19) showed that miR-223 was significantly increased in KD platelets. Consistently, our RT-PCR result also showed that the expression of platelet miR-223 was significantly increased in the KD group compared to the HC group **(** **Figure 1D** **)**. These results suggest that platelets and platelet-derived miR-223 may be involved in the pathophysiological process of KD.

### Activated KD platelets are internalized into HCAECs

**Figure 2:**
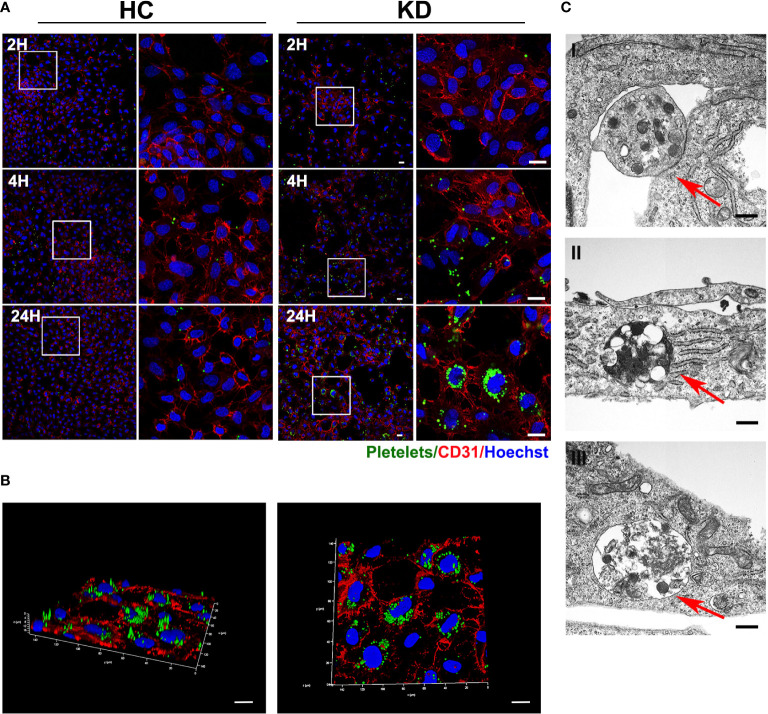
Activated KD platelets were internalized by HCAECs. **(A)** Representative immunofluorescence images of HCAECs co-cultured with CMFDA-labeled (green) HC or KD platelets for 2, 4, and 24 hours. Blue, Hoechst nuclear staining; red, CD31 in HCAECs (HC platelets: n = 6, KD platelets: n = 6). Scale bar: 20μm. **(B)** Confocal three-dimensional reconstruction of Z-stack images of HCAECs co-cultured with CMFDA-labeled KD platelets (green) for 24 hours (KD platelets: n = 6). Scale bar: 20μm. **(C)** Transmission electron microscopy of HCAECs co-cultured with KD platelets for 24h. (KD platelets: n = 3). I-III were images acquired in different fields of vision respectively. Red arrows indicated KD platelet internalized into HCAECs. Scale bar: 500nm.

As we recently demonstrated that activated KD platelets could be incorporated into VSMCs, we sought to investigate if KD platelets were internalized into ECs. HCAECs were co-cultured with CMFDA green-labeled platelets for 2, 4, and 24 hours. Consistent with our previous study that activated platelets endocytosed by VSMCs (19, 27), we found that activated KD platelets were also significantly incorporated into HCAECs (**Figure 2A**). Three-dimensional reconstruction had shown that KD platelets were considered at the same horizontal position as the nucleus of the HCAECs (**Figure 2B**). The incorporation of platelets by HCAECs was further confirmed by transmission electron microscopy (**Figure 2C** I-III and **Supplementary Figure 1**). Platelets were located at the membrane of HCAECs initially (**Figures 2C** I-red arrow), then incorporated into the cytoplasm (**Figures 2C** II-red arrow), and fused with lysosomes (**Figure 2C** III-red arrow). Taken together, these results indicate that activated KD platelets are internalized into HCAECs.

### The horizontal transfer of KD platelets derives miR-223 to HCAECs

**Figure 3:**
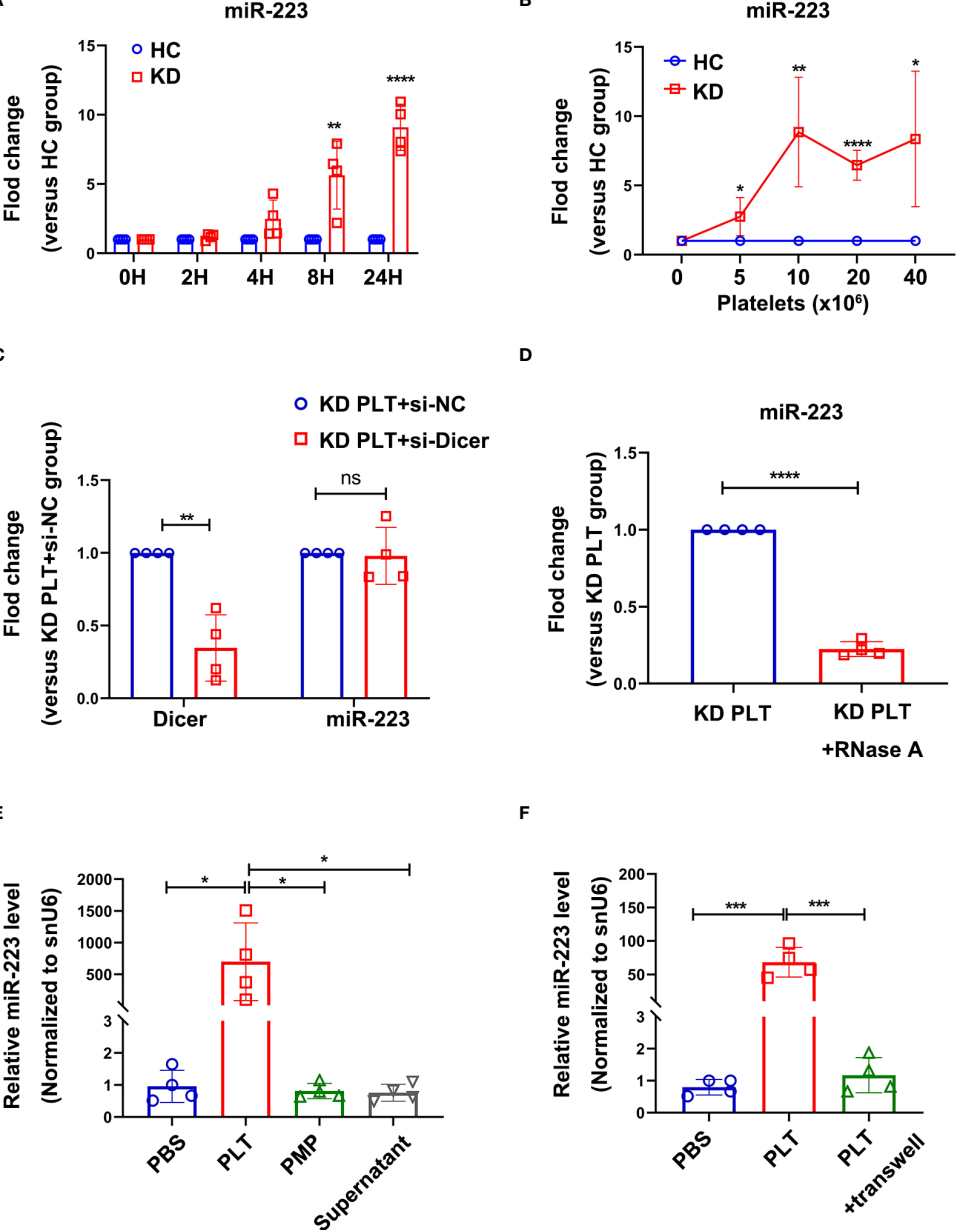
Intracellular miR-223 in HCAECs were delivered by activated KD platelets. **(A)** Level of miR-223 in HCAECs co-cultured with HC or KD platelets for 0, 2, 4, 8, and 24 hours. At each time point, the relative level of miR-223 in KD group was normalized by the HC group (n=4). Data are presented as mean ± SD, Unpaired *t-*test. **(B)** Level of miR-223 in HCAECs co-cultured with various number of platelets from HC or KD group for 24 hours. The relative level of miR-223 in KD group was normalized by each HC group (n=4). Data are presented as mean ± SD, Unpaired *t-*test. **(C)** The intracellular level of Dicer and miR-223 in HCAECs transfected with or without si-Dicer and subsequently co-cultured with KD platelets (1:100) for 24 hours. Results were presented as fold change (n=4). Data are presented as mean ± SD, Unpaired *t-*test. **(D)** The intracellular miR-223 level in HCAECs incubated with RNase A-treated (1U/ml, 1h, 37°C) KD platelets (1:100) for 24 hours. Results were presented as fold change (n=4), Unpaired *t-*test. Data are presented as mean ± SD. **(E)** The expression of miR-223 in HCAECs incubated with PLTs, PMPs, or supernatant, respectively for 24 hours (n=4). Data are presented as mean ± SD, One-way ANOVA and Tukey’s multiple comparisons test. **(F)** The expression of miR-223 in HCAECs incubated with KD platelets (PLTs) with or without a transwell chamber for 24 hours (n=4). Data are presented as mean ± SD, One-way ANOVA and Tukey’s multiple comparisons test. HC, HCAECs co-cultured with platelets isolated from HC; KD, HCAECs co-cultured with platelets isolated from KD patients. ^*^ *P*<0.05, ^**^ *P *< 0.01, ^***^ *P *< 0.001, ^****^ *P *< 0.0001. ns, no statistical difference.

Previous studies (31, 32) found that miR-223 was expressed in freshly isolated human vascular endothelial cells. However, the expression of miR-223 in ECs were gradually decreased until undetectable *in vitro* culture, suggesting for the exogenous source of miR-223 (31). Using THP-1 cells with high expression of miR-223 as a positive control, HCAECs were cultured with different FBS concentration or treated with VEGF, no significant expression of miR-223 was detected by RT-qPCR in HCAECs after five passages, *Ct* value >35 **(** **Supplementary Figure 2A** **)**. However, when co-cultured with KD platelets, the intracellular level of miR-223 in HCAECs increased with incubation time **(** **Figure 3A** **),** and the number of co-cultured platelets **(** **Figure 3B** **)**.

To determine if the platelet-derived miR-223 contributing to the increased level of miR-223 in HCAECs, the miR-223 expression level was first examined in HCAECs, suggesting a significantly increased level of miR-223 in HCAECs after co-culture with KD platelets **(** **Supplementary Figure 2B** **)**. The si-Dicer was transiently transfected into HCAECs to inhibit the splicing of pre-miRNA into mature miRNA by Dicer in HCAECs. HCAECs transfected with si-Dicer showed a significant reduction of Dicer mRNA (**Figure 3C**). When co-cultured with KD platelets for 24 hours, either HCAECs or si-Dicer transfected HCAECs showed no statistically significant difference in miR-223 **(** **Figure 3C** **)**. When co-cultured with RNase A-treated KD platelets for 24 hours, the level of miR-223 was significantly decreased in HCAECs **(** **Figure 3D** **)**. These results suggest that the horizontal transfer of platelet-derived miR-223 contributes to the increased level of miR-223 in HCAECs co-cultured with KD platelets.

Since platelets miRNAs can be directly released into the circulation or packaged in the PMPs. KD platelets, PMPs, and releasate from the same amount of blood were co-cultured with HCAECs for 24 hours respectively. HCAECs co-cultured with KD platelets showed significantly increased expression of miR-223. No significant difference in miR-223 level was found in HCAECs co-cultured with PMPs or supernatant containing platelet-releasate compared to the PBS group **(** **Figure 3E** **)**. Using purified KD platelets and a transwell chamber, we found significantly increased expression of miR-223 in HCAECs after directly incubated with KD platelets **(** **Figure 3F** **)**. These results suggest that the increased level of miR-233 in HCAECs co-cultured with KD platelets was mainly contributed by the platelet-transferred miR-223, rather than PMPs and free miRNA released by platelets.

### KD platelet-derived miR-223 attenuates monocytes adhesion to HCAECs

**Figure 4:**
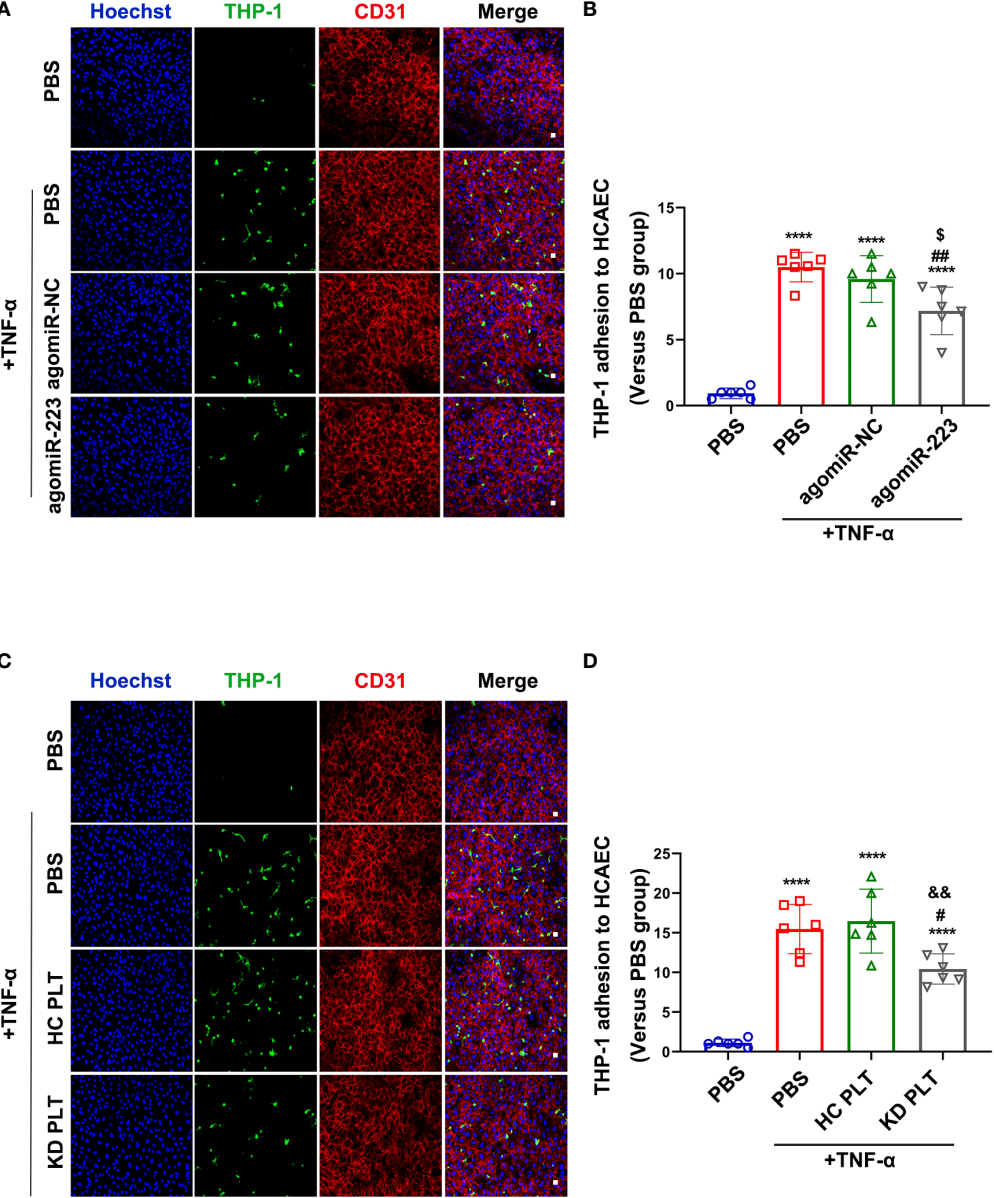
KD platelet-derived miR-223 inhibited monocyte adhesion to HCAECs. HCAECs were transfected with agomiR-223 or agomiR-NC for 48 hours followed by treatment with TNF-α (2ng/ml, 4h). **(A)** Representative immunofluorescence images of HCAECs co-cultured with CMFDA-labeled (green) THP-1 for 2 hours. Blue, Hoechst nuclear staining; red, CD31 in HCAECs (n = 6). Scale bar: 20μm. **(B)** The number of CMFDA-labeled THP-1 adhered to the surface of HCAECs. Results were presented as fold change(n = 6). Data are presented as mean ± SD, One-way ANOVA and Tukey’s multiple comparisons test. HCAECs were co-cultured with HC or KD platelets for 48 hours followed by treatment with TNF-α (2ng/ml, 4 hours). **(C)** Representative immunofluorescence images of HCAECs co-cultured with CMFDA-labeled (green) THP-1 for 2 hours. Blue, Hoechst nuclear staining; red, CD31 in HCAECs (n = 6). Scale bar: 20μm. **(D)** The number of CMFDA-labeled THP-1 adhered to the surface of the HCAECs. Results were presented as fold change (n = 6). Data are presented as mean ± SD, One-way ANOVA and Tukey’s multiple comparisons test. ^****^ *P *< 0.0001 vs. PBS group; ^#^ *P *< 0.05, ^##^ *P *< 0.01 vs. PBS+TNF-α group; ^$^ *P *< 0.05 vs. agomiR-NC+TNF-α group; ^&&^ *P *< 0.01 vs. HC PLT+TNF-α group.

Inflammatory factors are known to induce vascular endothelial cells inflammatory response by promoting leukocyte adhesion in vasculitis, e.g. TNF-α (33). Plasma TNF-α level was increased in patients with KD compared to healthy children **(** **Supplementary Figure 3** **)**. TNF-α treatment was used to mimic the inflammatory state of endothelial cells *in vitro*. To investigate the functional effect of increased miR-223 level in HCAECs, we performed leukocyte adhesion assay in TNF-α-activated HCAECs. THP-1 was used as a leukocyte representative. HCAECs were pre-transfected with agomiR-223 or agomiR-NC followed by incubation with THP-1 for 4 hours. The level of miR-223 was significantly increased after agomiR-223 transfection **(** **Supplementary Figure 4** **)**. Using fluorescence microscopy, we evaluated green fluorescence-labeled THP-1 on the surface of HCAECs. Treatment with TNF-α increased THP-1 adherence to the HCAECs, which was attenuated by approximately 30% when HCAECs over-expressing miR-223 **(** **Figures 4A, B** **)**. Adhesion of THP-1 to HCAECs induced by TNF-α showed no significant difference between HCAECs and HCAECs pre-cocultured with HC platelets. While pre-cocultured with KD platelets for 48h, the number of THP-1 adhesion to HCAECs showed a decreasing trend of about 40% **(** **Figures 4C, D** **)**. Our results demonstrate that KD platelet-derived miR-223 attenuates the adhesion of monocytes to HCAECs.

### KD Platelet-derived miR-223 targets ICAM-1 in HCAECs

**Figure 5:**
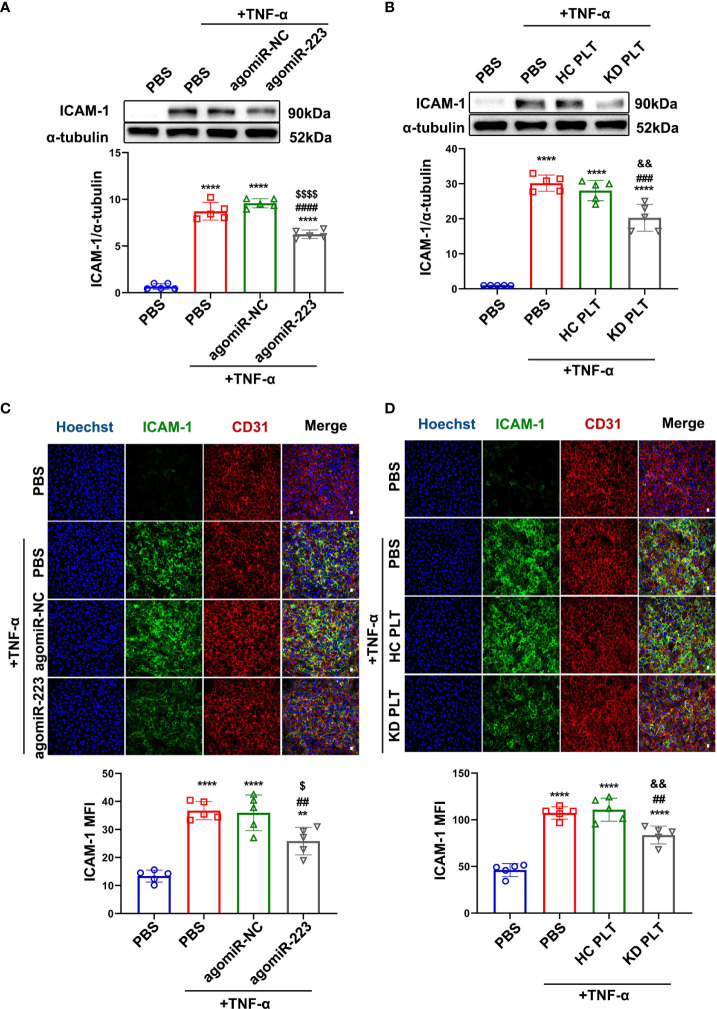
KD platelet-derived miR-223 attenuated TNF-α-induced ICAM-1 expression in HCAECs. **(A)** Expression of ICAM-1 in HCAECs transfected with agomiR-223/agomiR-NC for 48 hours followed by treatment with TNF-α (2ng/ml, 4 hours) was determined by Western blot analysis (n = 5). Data are presented as mean ± SD, One-way ANOVA and Tukey’s multiple comparisons test. **(B)** Expression of ICAM-1 in HCAECs and HCAECs co-cultured with HC or KD platelets for 48 hours followed by treatment with TNF-α (2ng/ml, 4 hours) was determined by Western blot analysis (n = 5). Data are presented as mean ± SD, One-way ANOVA and Tukey’s multiple comparisons test. **(C)** Representative immunofluorescence images and quantification of ICAM-1 in HCAECs transfected with agomiR-NC/agomiR-223 for 48 hours followed by TNF-α treatment. Green, ICAM-1; blue, Hoechst nuclear staining; red, CD31 in HCAECs (n = 5). Scale bar: 20μm. Data are presented as mean ± SD, One-way ANOVA and Tukey’s multiple comparisons test. **(D)** Representative immunofluorescence images and quantification of ICAM-1 in HCAECs and HCAECs co-cultured with HC or KD platelets for 48 hours followed by TNF-α treatment. Green, ICAM-1; blue, Hoechst nuclear staining; red, CD31 in HCAECs (n = 5). Scale bar: 20μm. Data are presented as mean ± SD, One-way ANOVA and Tukey’s multiple comparisons test. ^**^ *P *< 0.01, ^****^ *P *< 0.0001 vs. PBS group; ^##^ *P *< 0.01, ^###^ *P *< 0.001, ^####^ *P *< 0.0001 vs. PBS+TNF-α group; ^$^ *P *< 0.05, ^$$$$^ *P *< 0.0001 vs. agomiR-NC+TNF-α group; ^&&^ *P *< 0.01 vs. HC PLT+TNF-α group.

To identify the potential targets of miR-223 in HCAECs, miRNA target prediction software (TargetScan 8.0) was used. We found that ICAM-1 harbors a conserved binding site for miR-223 within its 3’ untranslated region (3’ UTR) **(** **Supplementary Figure 5A** **)**. ICAM-1 is a key endothelial receptor in the leukocyte-endothelial interaction and participates in the regulation of leukocyte adhesion to endothelial cells. A previous study using a dual-luciferase reporter assay has reported that ICAM-1 is a target gene of miR-223 in endothelial cells (29). To further determine if ICAM-1 is the target of miR-223, Ago2 immunoprecipitation was performed in HCAECs transfected with agomiR-223/agomiR-NC, and miR-223 and ICAM-1 mRNA were detected by RT-qPCR. The expression levels of miR-223 and ICAM-1 mRNA in the Ago2 complex extracted from HCAECs transfected with agomiR-223 were significantly increased, compared to cells transfected with agomiR-NC **(** **Supplementary Figure 5B** **)**. Next, we observed the regulation of miR-223 on ICAM-1 in endothelial cells under an inflammatory state. TNF-α treatment was used to mimic the inflammatory state of HCAECs as described above. Western-blot and immunofluorescence results indicated that TNF-α treatment increased the expression of ICAM-1 in HCAECs, which was suppressed by overexpression of miR-223 by approximately 35% **(** **Figures 5A, C** **)**. HCAECs were co-cultured with HC/KD platelets and then stimulated with TNF-α. We found that TNF-α-induced ICAM-1 expression was not significantly different between HCAECs and HCAECs pre-cocultured with HC platelets. But, the protein expression of ICAM-1 was reduced by about 30% in HCAECs pre-cocultured with KD platelets **(** **Figures 5B, D** **)**. Taken together, our results demonstrate that KD platelet-derived miR-223 targets ICAM-1 in HCAECs.

### In the LCWE-induced KD murine model, down-regulation of platelet miR-223 increases endothelial ICAM-1 expression with concomitant infiltration of inflammatory cells in abdominal aorta

**Figure 6:**
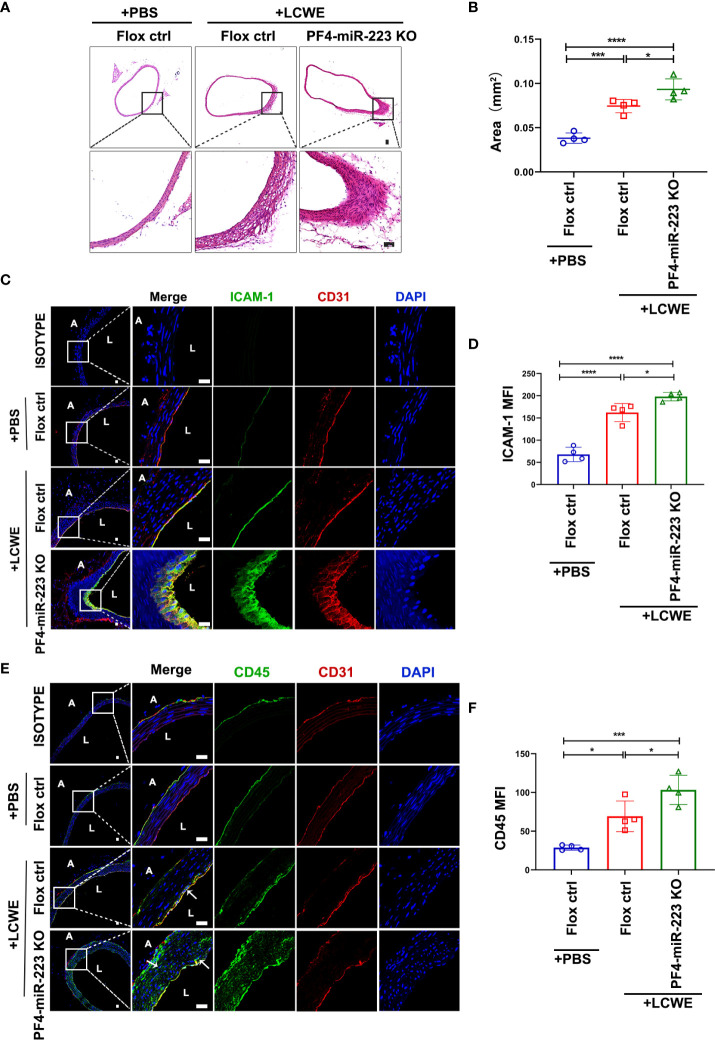
In LCWE-induced KD murine model, deficiency of platelet-miR-223 exacerbated the medial thickening, increased ICAM-1 expression with concomitant CD45^+^ inflammatory cells infiltration in the endothelium of abdominal aorta. LCWE or PBS was administrated *i.p.* for two weeks. The abdominal aorta tissues were collected, and continuous cross-sections were performed for H&E staining and immunofluorescence analysis. **(A)** Representative H & E-stained sections of PBS or LCWE injected mice were shown. n = 4, Scale bar: 50μm. **(B)** Quantification of the media layer areas of abdominal aorta in each group was shown. n = 4, Data are presented as mean ± SD, One-way ANOVA and Tukey’s multiple comparisons test. **(C)** Representative immunofluorescence images of sections from PBS or LCWE-injected mice. CD31 was stained as red, ICAM-1 as green, and the nucleus visualized as blue (DAPI). n = 4, Scale bars: 20μm. **(D)** Quantification of ICAM-1 expression in abdominal aorta tissues was shown (n = 4). Data are presented as mean ± SD, One-way ANOVA and Tukey’s multiple comparisons test. **(E)** Representative immunofluorescence images of sections from PBS or LCWE-injected mice. CD31 was stained as red, CD45 as green, and the nucleus visualized as blue (DAPI). n = 4, Scale bars: 20μm. **(F)** Quantification of CD45 expression in abdominal aorta tissues was shown (n = 4). Data are presented as mean ± SD, One-way ANOVA and Tukey’s multiple comparisons test. L, lumen; A, adventitia; Flox ctrl: miR-223 ^flox/flox^ mice.PF4-miR-223 KO, PF4-cre: miR-223 ^flox/flox^ mice. ^*^ *P *< 0.05, ^***^ *P *< 0.001, ^****^ *P *< 0.0001.

To determine the role of platelet-derived miR-223 to KD vasculitis *in vivo*, the platelet-specific miR-223 knockout mice (PF4-miR-223 KO, PF4-cre: miR-223^flox/flox^) were successfully constructed, LCWE was intraperitoneally injected (*i.p*) to establish KD murine model. **Supplementary Figure 6A** demonstrated the generation of a recombined allele upon Cre recombination in generated mice. RT-qPCR results showed that miR-223 expression in platelets was significantly decreased in PF4-miR-223 KO mice **(** **Supplementary Figure 6B** **)**. Two weeks after LCWE injection, the murine abdominal aorta was characterized by disruption of elastin, and manifestations of medial thickening, which were significantly enhanced in the PF4-miR-223 KO mice compared to Floxed ctrl mice **(** **Figures 6A, B** **)**. Our immunofluorescence results showed that the expression of ICAM-1 was significantly increased in the endothelium of the abdominal aorta after LCWE treatment. LCWE-injected PF4-miR-223 KO mice exhibited increased ICAM-1 expression compared to LCWE-injected Floxed ctrl mice **(** **Figures 6C, D** **)**. Consistently, we found the infiltration of CD45^+^ inflammatory cells was increased in the abdominal aorta of LCWE-injected KD murine model. Compared to LCWE-injected Floxed ctrl mice, LCWE-injected PF4-miR-223 KO mice exhibited increased CD45^+^ inflammatory cells infiltration **(** **Figures 6E, F** **)**. These results suggest that platelet-derived miR-223 inhibits endothelial ICAM-1 expression and the inflammatory cells infiltration in KD vasculopathy.

## Discussion

Kawasaki disease is a childhood-acquired cardiovascular disease characterized by multi-systemic vasculitis, accompanied by a series of inflammatory responses, including inflammatory cell activation, platelet hyperactivation, and dysfunction of the endothelial cells. Necrosis of endothelial cells in the early stages of KD is critical to disturbance of the vascular barrier (5). Platelets have been found to contribute to the development of endothelial dysfunction (34). Studies have focused on the effect of platelet-derived cytokines on the formation of endothelial lesions. Here we demonstrate that KD platelet-derived miR-223 inhibits the endothelial expression of ICAM-1, at least in part contributing to attenuating the adhesion of inflammatory cells (e.g., monocytes) in KD vasculitis.

In recent years, it has been found that platelet-derived miRNAs can be released into the peripheral blood *via* encapsulation in PMPs (35). In contact with the neighboring cells, the internalized PMPs deliver miRNAs, which regulate the function of the recipient cell by post-transcriptional reprogramming (36). MiR-223, which is abundantly expressed in human platelets, is encapsulated in PMPs and released upon thrombin stimulation (28). Co-incubation of HUVECs with PMPs increased the level of miR-223 in HUVECs and inhibited IGF-1R expression, which promoted the endothelial cell apoptosis after incubation with late glycosylation end products (26). In addition, platelet internalization was recently identified as a novel pathway for miRNA delivery (19, 27, 37). It has been reported that platelets were internalized into the hepatocyte. Following this internalization, platelets transfer RNA to the hepatocyte, which stimulated hepatocyte proliferation (37). Our recent study has demonstrated that the horizontal transfer of platelet-derived miR-223 increased the level of miR-223 in VSMCs, which suppressed VSMC dedifferentiation by inhibiting platelet-derived growth factor receptor β (PDGFRβ) in VSMCs (27). Consistently, we found platelets from KD patients with coronary aneurysms showed a significant decrease in miR-223 expression, compared with platelets from KD patients without coronary aneurysms (19). Deficiency of platelet miR-223 in KD patients with aneurysms contributes to coronary artery pathology as platelet uptake fails to suppress VSMC dedifferentiation. Thus, platelet-derived miR-223 was identified as a protective role in KD-induced arterial aneurysms. In the present study, our results showed that KD platelets with a higher level of miR-223 were incorporated into HCAECs, resulting in the horizontal transfer of miR-223. Using KD platelets, PMPs, and platelet-releasate from the same amount of blood co-cultured with HCAECs, we found the increased expression of miR-223 in HCAECs was primarily derived from KD platelets, rather than PMPs or free miRNAs from platelet releasate. Since miR-223 targets ICAM-1, the horizontal transfer of platelet-derived miR-223 contributes to the downregulation of ICAM-1 in HCAECs. Our results provide evidence for the regulatory function of horizontal transfer of platelet-derived miRNAs.

It is well known that ICAM-1 is a key endothelial receptor contributing to leukocyte-EC interactions and plays a critical role in inflammatory responses (38, 39). Adhesion of leukocytes, particularly monocyte adhesion and migration to ECs, is critical for the development of vasculitis (40). ICAM-1 is generally expressed at low basal levels in endothelial cells, but its expression is upregulated by inflammatory cytokines (41). Clinical studies have shown that the circulating levels of ICAM-1 expression are increased in the KD and positively correlated with disease severity (42, 43), but the underlying mechanism remains elusive. ICAM-1 was identified as one of the target genes of miR-223 (29). Studies on sepsis showed that TNF-α induced expression of ICAM-1 in endothelial cells was partially attenuated by miR-223 transferred by PMPs (25). However, studies on the involvement of miR-223 regulating ICAM-1 in endothelial cells were limited to *in vitro* studies (25, 44). Our results show that KD platelet-derived miR-223 also attenuates TNF-α induced expression of ICAM-1 in HCAECs. In LCWE-injected PF4-miR-223 KO mice, deficiency of platelet-miR-223 exacerbates the medial thickening of the abdominal aorta, increased ICAM-1 expression with concomitant CD45^+^ inflammatory cells infiltration into the endothelium compared to LCWE-injected Floxed ctrl mice, suggesting that platelet-derived miR-223 play an important role in endothelial pathology of KD.

There are some limitations in this study. First, platelet-derived miR-223 only partially attenuates TNF-α induced expression of ICAM-1, suggesting that other mechanisms may be involved. Wang et al. demonstrated that miR-223 exerted a protective effect by targeting IL6ST in KD-induced endothelial injury (45). Another study showed that miR-223 regulated EC proliferation by targeting IGF-1R in KD (31). Furthermore, Maruyama et al. proposed that miR-223 plays an important role in KD vasculitis by targeting NLRP3 (46). These studies suggest that miR-223 plays an important protective role in KD vasculitis. Our study demonstrates that platelet-derived miR-223 at least in part contributes to KD vasculopathy by targeting ICAM-1. Second, in PF4-miR-223 KO mice, miR-223 expression in platelets was decreased by 80%, in comparison with those from Floxed ctrl mice. We think impure platelet preparation may contribute to the incomplete knockout of miR-223 in platelets. Third, due to the limitation amount of blood samples, not all patient plasma were used for ELISA detection; Forth, in our recent study (19), TNF-α was detected to induce the expression of miR-223 *via* regulating the activity of Dicer, which further cleaves pre-miR-223 into mature miR-223 (47), suggesting the important role of TNF-α in KD platelet hyperreactivity. Other pro-inflammatory cytokines, such as interleukin-1β (IL-1β), were also found to contribute to KD vasculopathy (48), so further studies are warranted to explore the role of platelet-derived miR-223 in IL-1β induced vasculopathy.

**Figure 7:**
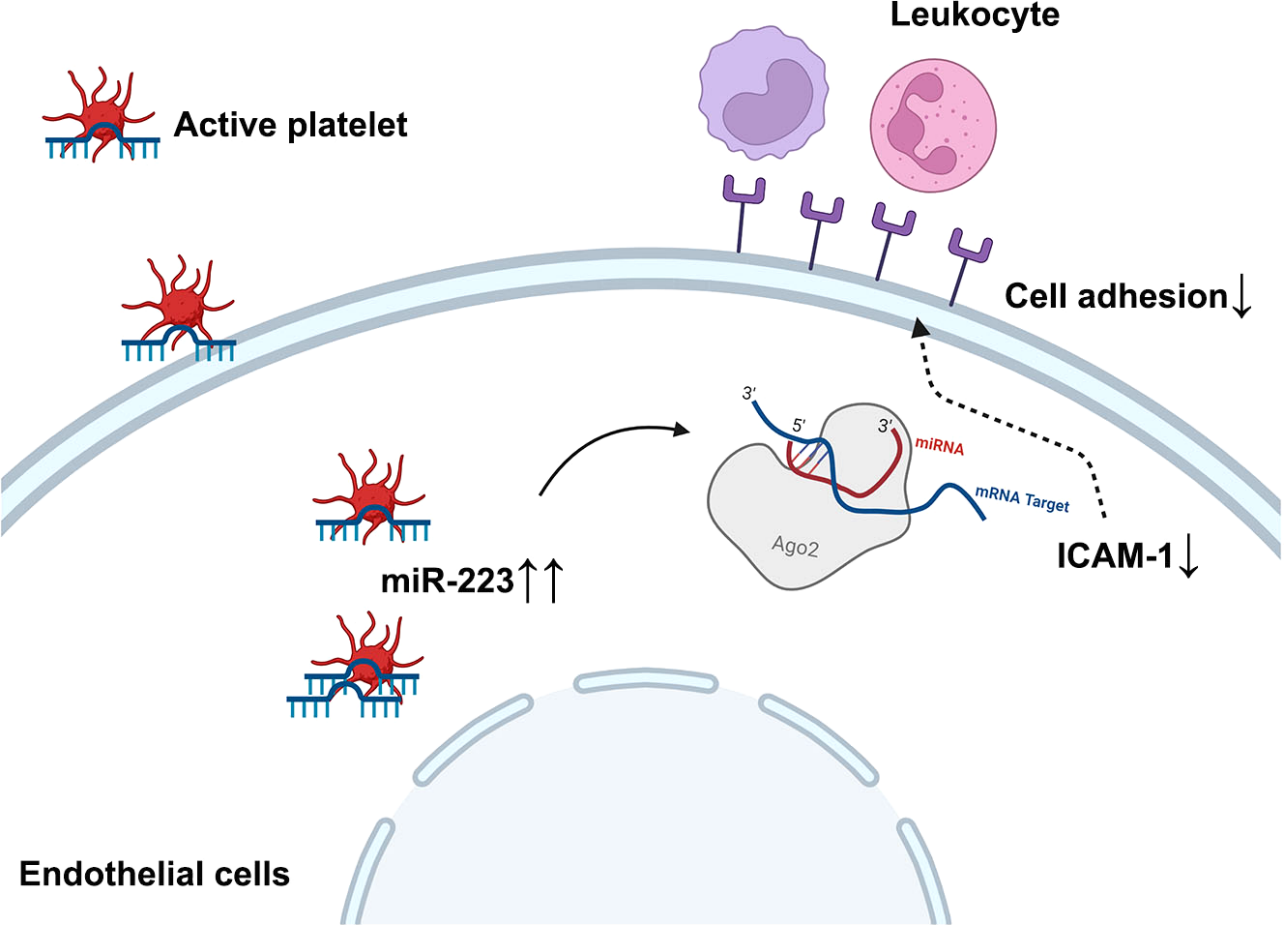
Schematic figure of the working model diagram of the regulatory function of platelet-derived miR-223 in endothelial cells. In the case of KD, platelets are hyperactive and internalized into HCAECs. The horizontal transfer of platelet derives miR-223 to HCAECs, which suppress the expression of ICAM-1 in HCAECs and partially attenuates leukocyte adhesion, thereby reducing further endothelial damage in KD vasculitis. ICAM-1, intercellular cell adhesion molecule-1.

In summary, we demonstrate that the horizontal transfer of platelet-derived miR-223 suppresses the expression of ICAM-1 in HCAECs, which at least in part attenuates leukocyte adhesion, thereby reducing endothelial damage in KD vasculitis (**Figure 7**). Platelet-derived miR-223 may serve as a brake for inflammatory cell infiltration into the endothelium. Thus, miR-223 level in platelets may serve as a potential prognostic marker for KD vasculopathy.

## Data availability statement

The original contributions presented in the study are included in the article/**supplementary material**. Further inquiries can be directed to the corresponding authors.

## Ethics statement

The studies involving human participants were reviewed and approved by Guangzhou Women and Children Medical Center Human Investigation Committee, No. 2017102710. Written informed consent to participate in this study was provided by the participants’ legal guardian/next of kin. The animal study was reviewed and approved by Animal Care and Use Committee of Guangzhou Medical University, China (2019-384).

## Author contributions

MG and YZ conceptualized this study. MG, SF, QC, CJ, MQ, YB, YZ, and WT determined the methodology. MG, SF, QC, CJ, and YZ performed the investigations. MG and YZ wrote the original draft. SF, MQ, YB, and WT wrote, reviewed, and edited the draft. YZ and WT supervised the overall study. All authors contributed to the article and approved the submitted version.

## Funding

This work was supported by the National Natural Science Foundation of China (Grant No. 81902144, 82022033, 81970437, 81903605, 31900527), the Ministry of Science and Technology of the People’s Republic of China (Grant No. QN20200131001), the Guangzhou science and technology project (Grant No. 201904010483), and Guangzhou Medical University (Grant No. 02-410-2206047).

## Conflict of interest

The authors declare that the research was conducted in the absence of any commercial or financial relationships that could be construed as a potential conflict of interest.

## Publisher’s note

All claims expressed in this article are solely those of the authors and do not necessarily represent those of their affiliated organizations, or those of the publisher, the editors and the reviewers. Any product that may be evaluated in this article, or claim that may be made by its manufacturer, is not guaranteed or endorsed by the publisher.
