# Supplementary material for: Platelet-derived microRNA-223 attenuates TNF-α induced monocytes adhesion to arterial endothelium by targeting ICAM-1 in Kawasaki disease
Source: Front Immunol. 2022 Aug 2;13:922868. doi: 10.3389/fimmu.2022.922868 (PMC9379370; doi:10.3389/fimmu.2022.922868)
Supplement: Supplementary file 1 [file DataSheet_1.docx]

Supplementary Material

# Supplementary Figures and Tables

**Supplementary Table 1:** The sequences for miRNA mimic.

| Gene | sense (5’-3’) | antisense (5’-3’) |
| --- | --- | --- |
| AgomiR-NC | UUCUCCGAACGUGUCACGUTT | ACGUGACACGUUCGGAGAATT |
| AgomiR-223 | UGUCAGUUUUGUCAAAUACCCA | GGGUAUUUGACAAACUGACAUU |
| si-Dicer1 | GUGCCGUAUUGGUAGUUCUTT | AGAACUACCAAUACGGCACTT |

**Supplementary Table 2:** The primer sequences used in RT-qPCR.

| Primer | Forward sequence | Reverse sequence |
| --- | --- | --- |
| Has-miR-223 | GTTGCTCCTGTCAGTTTGTCAAA | TATGGTTGTTCACGACTCCTTCAC |
| mus-miR-223 | GTTGCTCCTGTCAGTTTGTCAAA | TATGGTTGTTCACGACTCCTTCAC |
| snU6 | ATTGGAACGATACAGAGAAGATT | GGAACGCTTCACGAATTTG |
| ICAM-1 | ATGCCCAGACATCTGTGTCC | GGGGTCTCTATGCCCAACAA |
| Dicer | TGCTATGTCGCCTTGAATGTT | AATTTCTCGATAGGGGTGGTCTA |
| GAPDH | CTGGGCTACACTGAGCACC | AAGTGGTCGTTGAGGGCAATG |

## Supplementary Table 3: The primer sequences used in PCR.

| Primer | Forward sequence | Reverse sequence |
| --- | --- | --- |
| miR-223 Flox | GCATACAAGAAGTTGATAGGAGGAAG | GACTAAGACACACACCAAAAGAGGGAA |
| PF4 CRE | CCAAGTCCTACTGTTTCTCACTC | TGCACAGTCAGCAGGTT |

## Supplementary Figures


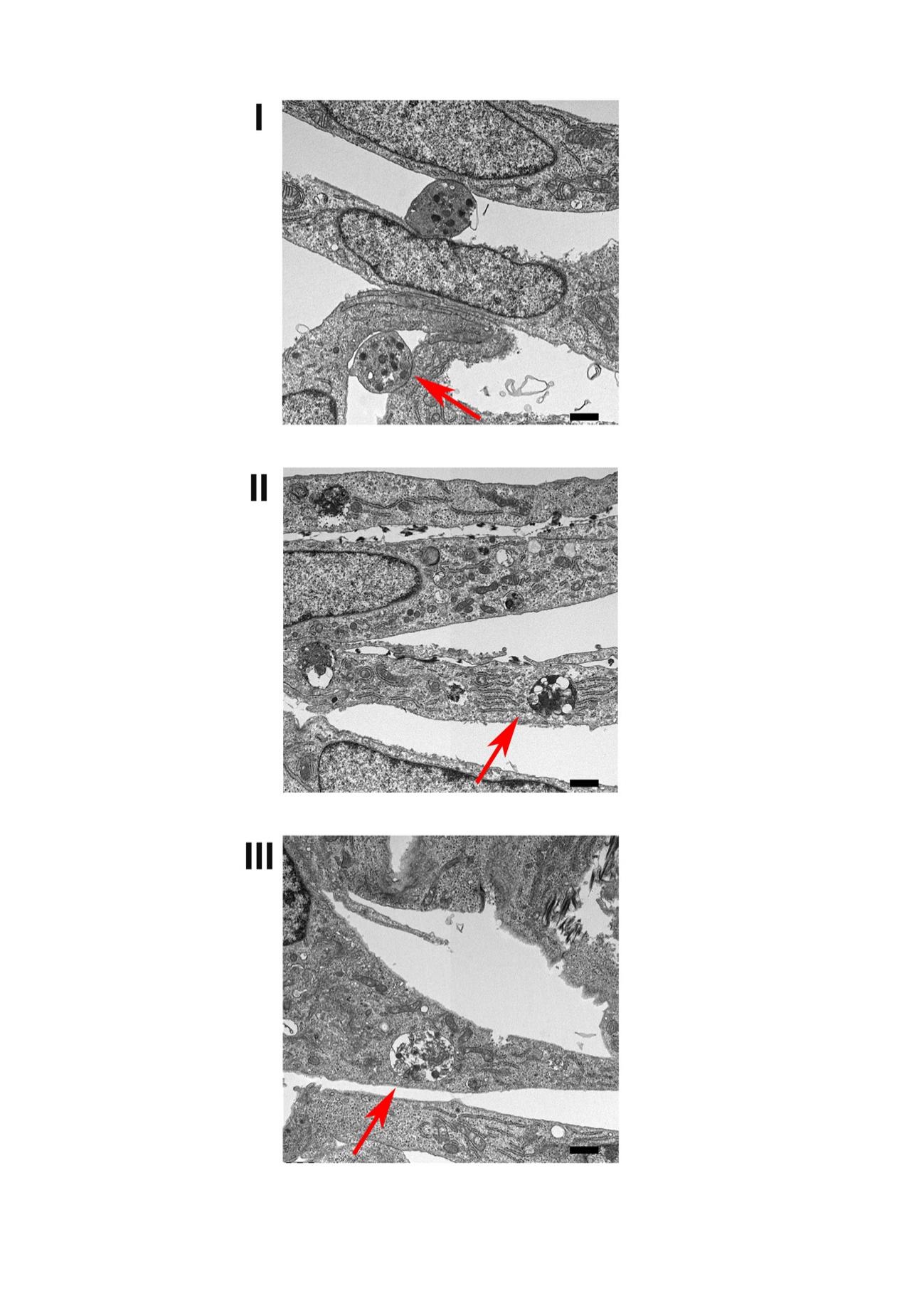


**Supplementary Figure 1.** Transmission electron microscopy of HCAECs co-cultured with KD platelets for 24 hours.

Lower magnification of each micrograph of KD platelets internalized into HCAECs after 24 hours of incubation was taken by transmission electron microscopy. I-III were images acquired in different fields of vision respectively. Red arrows indicated KD platelet internalized into HCAECs. KD platelet: n=3, Scale bar: 1μm.


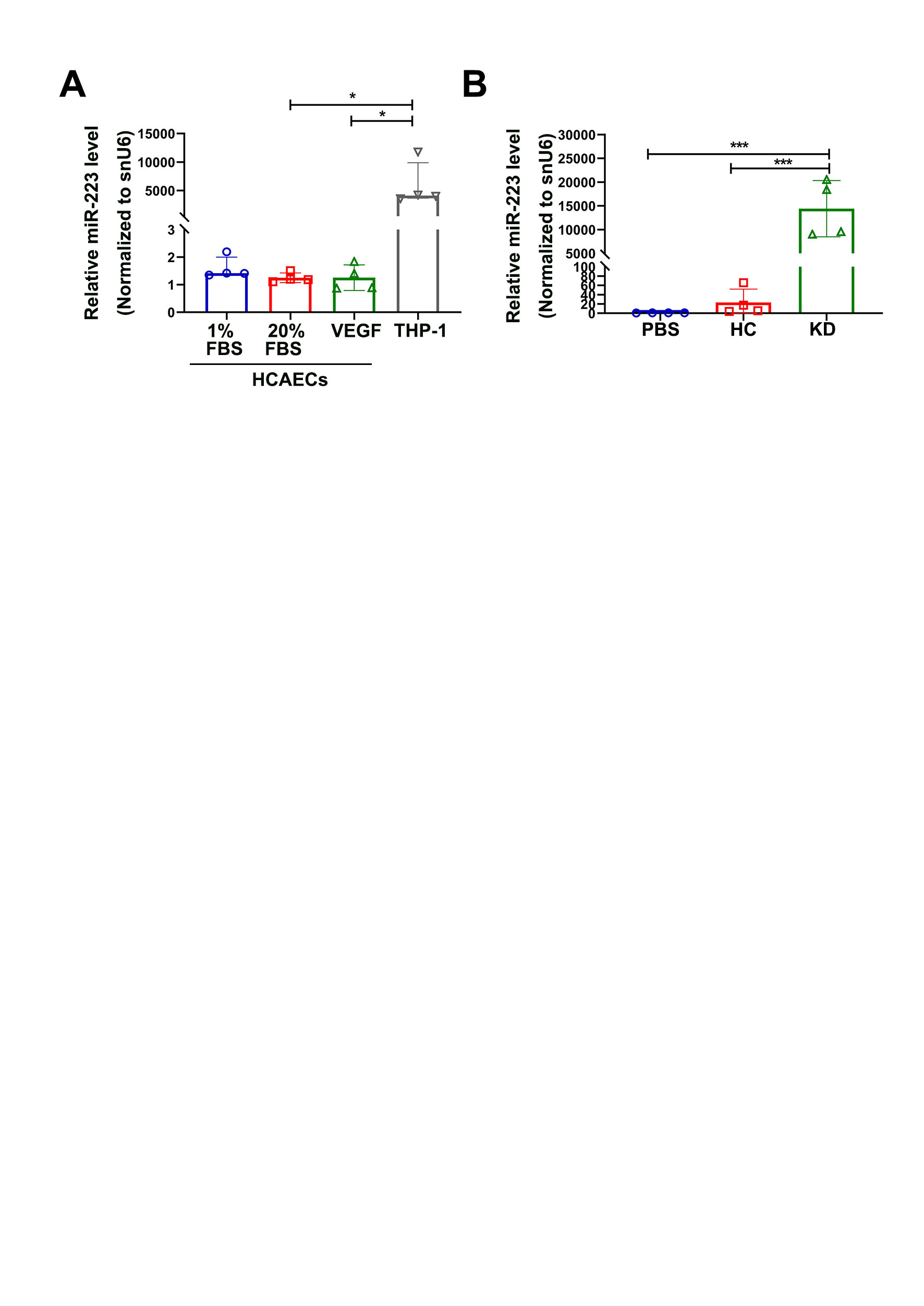


**Supplementary Figure 2.** Incubation with KD platelets increased intracellular level of miR-223 in HCAECs.

**(A)**The expression of miR-223 in HCAECs cultured in different FBS concentrations, or with vascular endothelial cell growth factor (VEGF; 30ng/mL) for 24 hours. The THP-1 cells with high level of endogenous miR-223 were used as positive control (n=4). Data are presented as median ± IQR, Kruskal-Wallis test and Dunn's multiple comparisons test. **(B)** The intracellular miR-223 level of HCAECs after incubation with or without platelets (1:100) for 24 hours (n=4). Data are presented as mean ± SD, One-way ANOVA and Tukey's multiple comparisons test.


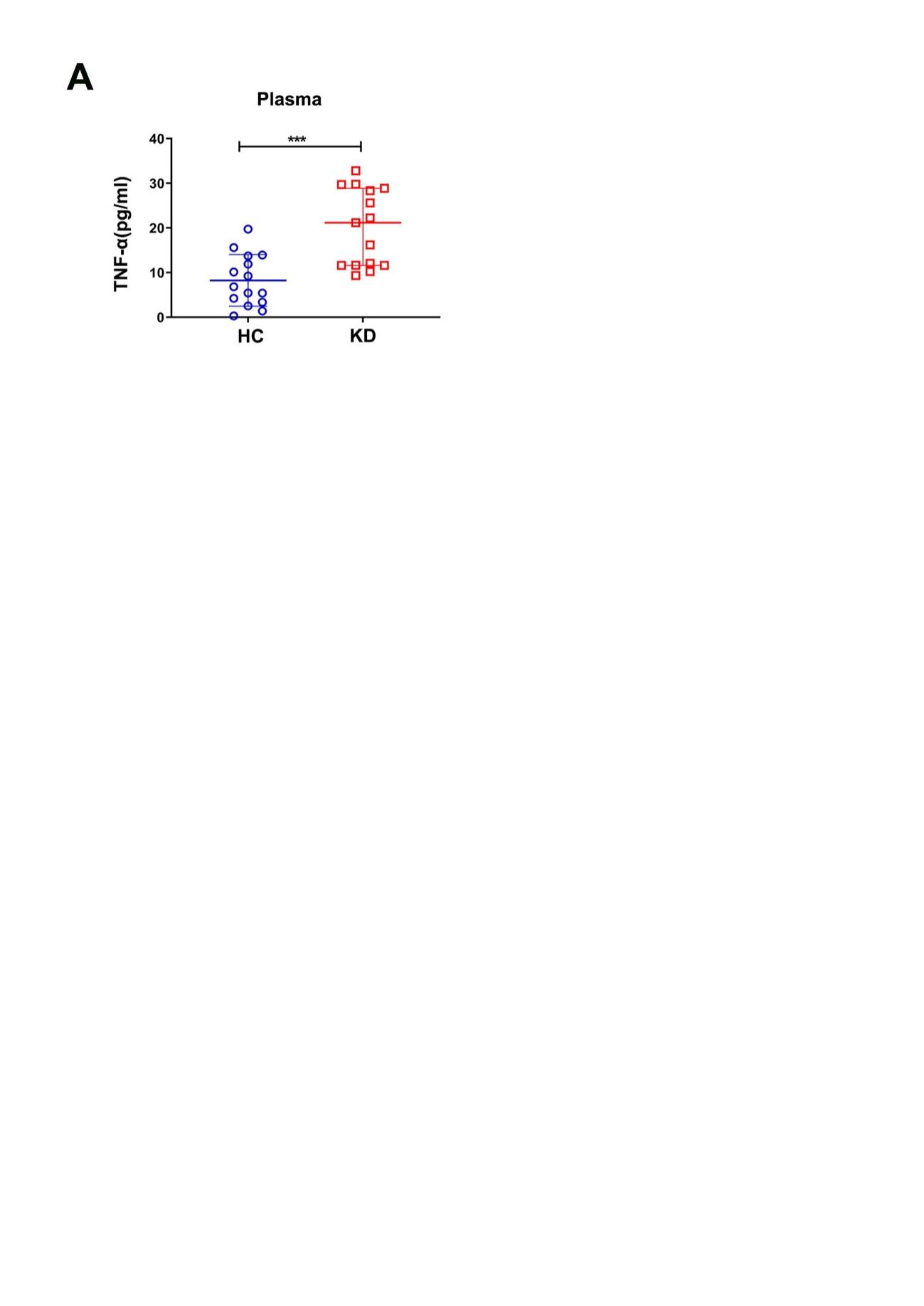


**Supplementary Figure 3.** Plasma TNF-α level was increased in patients with KD.

Plasma level of TNF-α in HC and KD group (HC: n=15, KD: n=15). Data are presented as median ± IQR, Mann Whitney test. ^***^*P*<0.001.


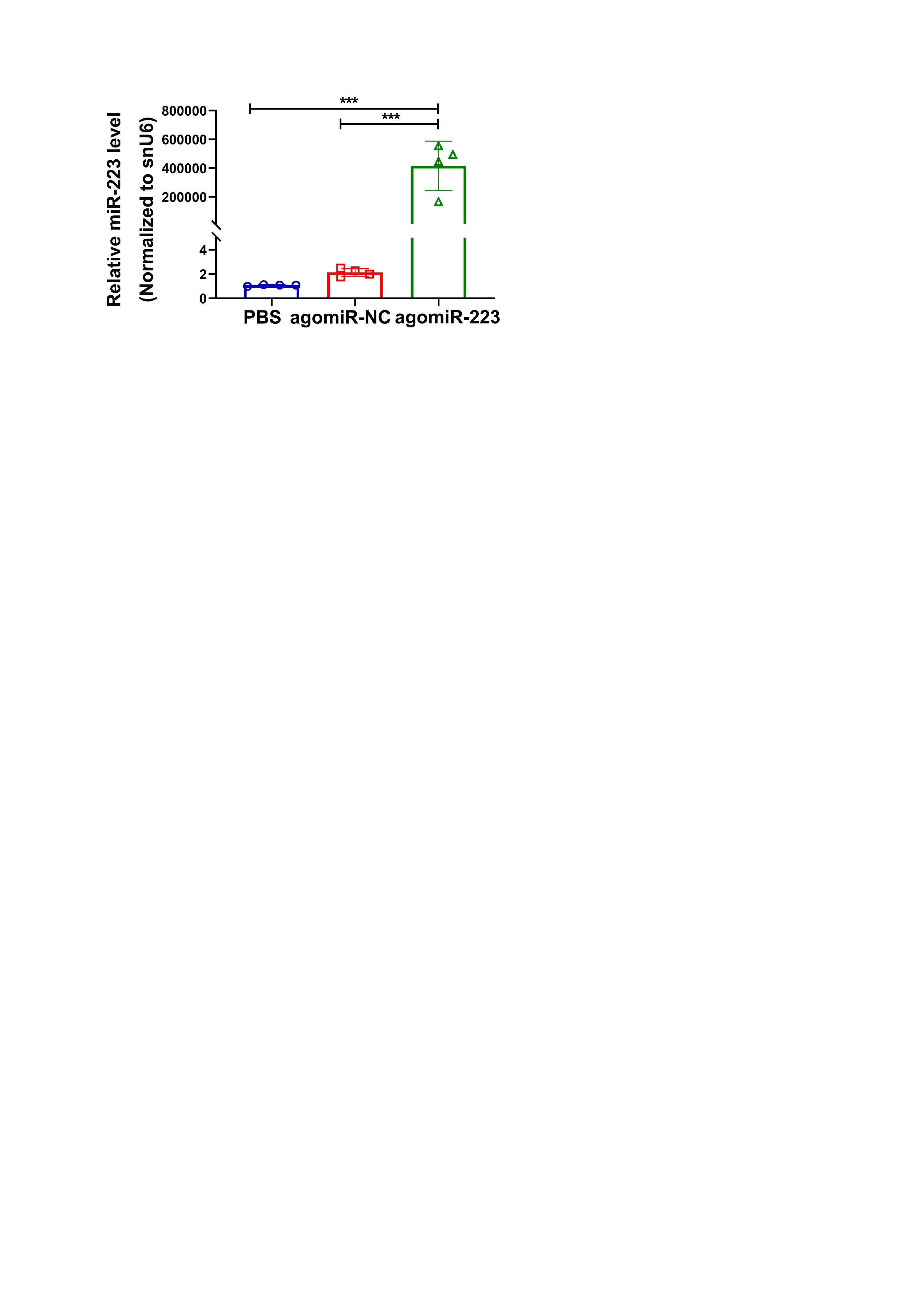


**Supplementary Figure 4.** Transfection with agomiR-223 increased intracellular level of miR-223 in HCAECs.

The intracellular miR-223 level of HCAECs transfected with agomiR-NC/agomiR-223 for 24 hours (n=4). Data are presented as mean ± SD, One-way ANOVA and Tukey's multiple comparisons test. ^***^*P*<0.001.


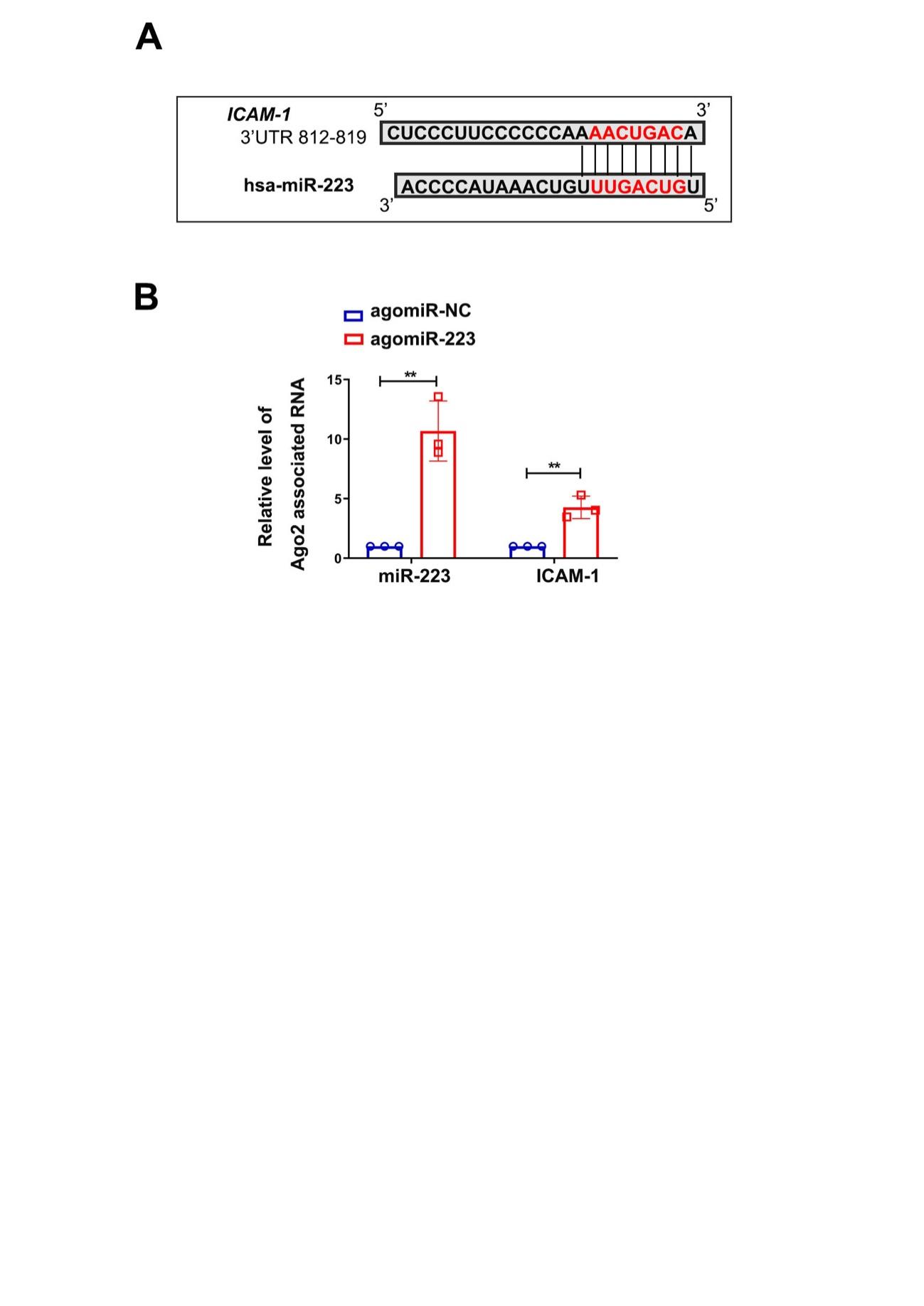


**Supplementary Figure 5.** ICAM-1 is a target gene of miR-223 in endothelial cells.

**(A)**Predicted miR-223 target site within 3′ untranslated region (3′ UTR) of ICAM-1(mRNA) gene by TargetScan8.0 bioinformatic analysis. **(B)**Ago2 immunoprecipitation was performed in HCAECs transfected with agomiR-223/agomiR-NC. Ago2-related miR-223 and ICAM-1 mRNA levels were quantified (n=3). Data are presented as mean ± SD, Unpaired *t-*test. ^**^*P*<0.01.


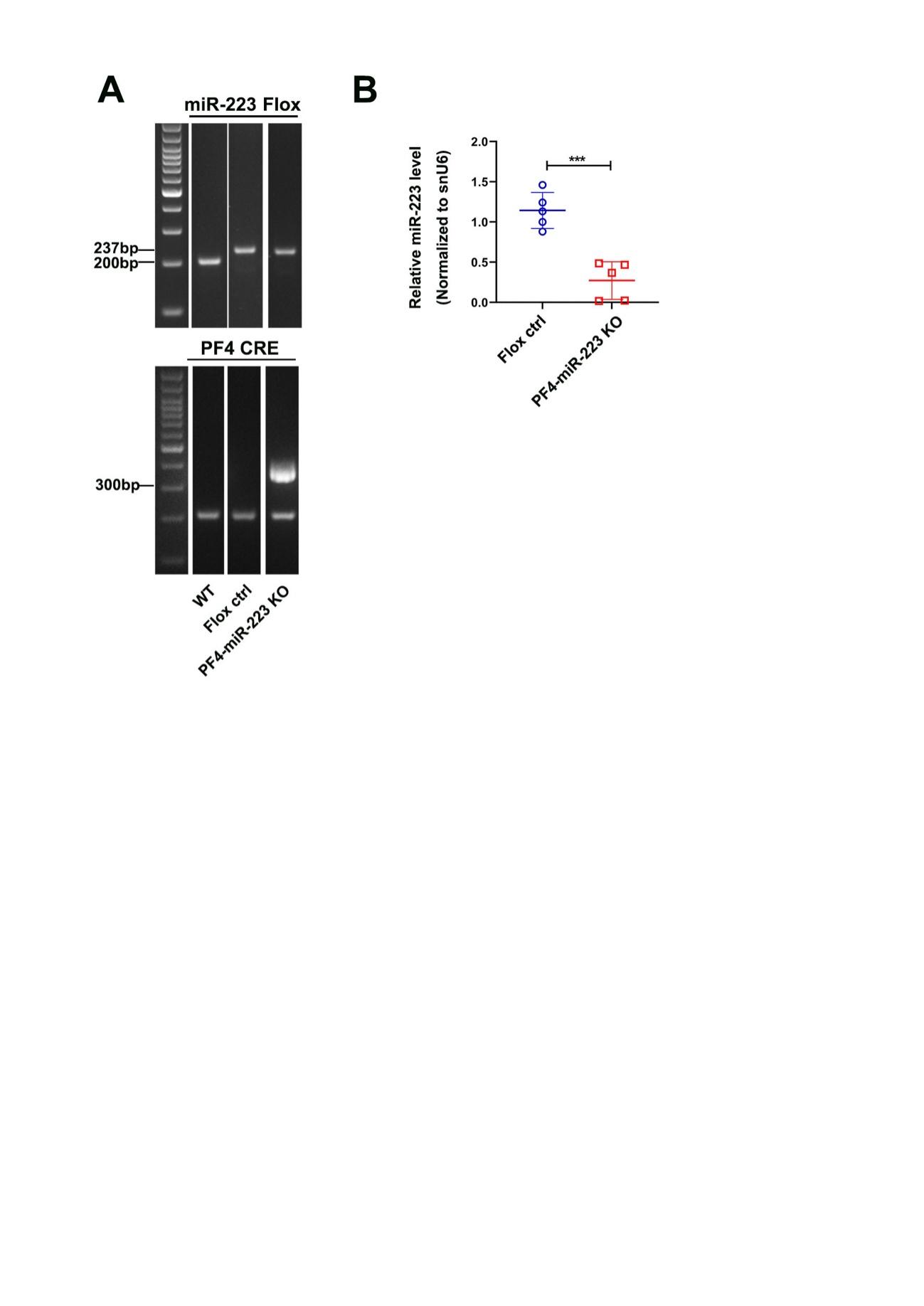


**Supplementary Figure 6.** Platelet miR-223 is significantly decreased in PF4-miR-223 KO mice.

**(A)**Verification of PF4 cre -mediated excision using genomic PCR. **(B)** Relative expression of miR-223 in platelets from PF4 miR-223 KO mice and Floxed ctrl mice (n=5). Data are presented as mean ± SD, Unpaired *t-*test. Abbreviations: Flox ctrl: miR-223 ^flox/flox^ mice .PF4-miR-223 KO, PF4-cre: miR-223 ^flox/flox^ mice. ^***^*P*<0.001.
